# Our Correspondence

**Published:** 1869-07

**Authors:** 


### Our Correspondence.

**Six Interesting Letters with Answers.**

SAVANNAH, Ga., May 25, 1869.

THAD. S. UP DE GRAFF, M. D.,

*Dear Sir*:—Inclosed find P. O. order for $15, for club of Fifty, for your excellent Magazine, the BISTOURY. I never earned ten dollars easier, and expect to send you another club before next issue.

Now let me tell you how I have been swindled by those outrageous humbugs whom you have so frequently exposed in the BISTOURY. Had I not read your article, explaining their way of doing things, I suppose I would still be sending them my money.

I first sent that "Dr. Ogden" of New York fifteen dollars at different times for his "medicine already prepared," for spermatorrhœa or seminal weakness. It did me no good, but what is worse, a chemist to whom I submitted this medicine (?) informed me that it is *inert*—absolutely *worthless*, the largest cost in preparing it being the bottle and cork. I next sent money to the "Howard Association," of Philadelphia, but soon learned that the "Association" consisted of one man, and *he* not a physician. So I quit in that direction. In short, I tried every humbug who advertises in the daily papers of every city, and who agree to send a formula *free* to any one afflicted as I am, and every confounded rascal of them managed to get from $5 to $15 out of me for their worthless medicines. How long I should have kept this up I know not, had I not come in contact with the BISTOURY, and learned how these fellows thrive upon the earnings of swindled young men like myself. I must thank you, Doctor, for the kind letter of advice written me, and am happy to say that I am finally getting well through following your directions.

I write this to warn other unfortunate young men to avoid the advertised nostrums for spermatorrhœa. I have tried them thoroughly, and *know* they are *worthless*.

Very truly and gratefully yours,

J. R. N.

—This is a fair sample of more than one dozen letters that we have received from all over the country, from young men who have been swindled out of their money by the scoundrels mentioned in his letter. His experience may serve to warn others against such impositions, and induce persons so afflicted to consult regularly educated physicians for advice in such matters.

---

COLUMBUS, Ga., April 4, 1869.

DR. T. S. UP DE GRAFF:

*Dear Sir*:—While waiting in the office of my physician to-day for his arrival, I picked up a copy of your journal, and being attracted by your answers to correspondents, have determined to ask you one question.

My daughter, though having a finely shaped head and forehead, is annoyed by a growth of fine *dark* hair on the temples and the top of the forehead, below the natural hair. It will not grow long enough to brush in with the other hair, rendering it impossible to dress it in the prevailing mode, and disfiguring an otherwise handsome forehead. I have tried Depilatories, but they afford only temporary relief. Extraction with tweezers, the same. My doctor tells me that the gland not being destroyed, of course the hair will grow.

Now, Sir, it seems to me that if after the hair was pulled out, while the orifice was still open, that if something could be rubbed in that would inflame and destroy the gland, the object would be attained. Can it not be done? Please answer in your next BISTOURY, and I will ask the Doctor to look out for the number.

Yours very truly, W.

Answer:—Depilatories should be used with great caution, and then only under the direction and care of a physician. The following compound has always succeeded in our hands. If it is properly and carefully applied it will give you the desired result.

Take orpiment, ¼ oz.

Quick Lime, 1 oz.

Starch, ½ oz.

Rub well together, and add water sufficient to form a soft paste. Spread on the parts from which the hair is to be removed, and when nearly dry, wash off. Dress with sweet oil, and pluck the hair out with tweezers, one by one.

---

MACHIAS, April 22, 1869.

Cataraugus County, N. Y.

*Thad. S. Up De Graff, M. D.*, DEAR SIR:—Enclosed you will find fifty cents, to pay for the BISTOURY one year. I am 72 years old, almost deaf. I have been humbugged. I have had letters sent me from different places, stating how they could cure me of my deafness. I sent them money—got swindled. One man from Philadelphia, (Ramsey, by name,) sent me a pamphlet of certificates, (all penned by one hand,) telling of wondrous cures, (all a humbug, of course.) If I would send him five dollars he would send me a prescription. I sent the money. He sent me two very small vials of some harmless stuff; and if that didn't cure I was to send five dollars more. He stated that some had to send three or four times. But I sent no more money to him, but got swindled, you know. Some traveling quack would come along, could surely cure. I pay the money, and get swindled again.

I will tell no more, for you will think I am a dunce. Now what ought to be done with such Doctors, or quacks? State Prison is too good for them. Hang them; send them to the d—l at once! I do not expect to be cured.

Yours, &c., W. M. F.

—We judge from the conclusion of the old gentleman's letter that he is considerably disgusted with the Quacks of the day. We publish his letter for the benefit of others.

---

WAVERLY, Tioga County, N. Y., April 5, 1869.

T. S. UP DE GRAFF, M. D.:

*Dear Sir:*—Your BISTOURY fell into my hands some time ago, and I have been thinking that you could prescribe for my disease. I entered the army in July, 1863, was wounded in May, '64, and had my arm amputated. It healed, and remained so for about one year. It then broke out and has continued to discharge from two places since. Three pieces of bone came out about two years ago. If you know of any remedy I would be very thankful if you would send it to me. Very respectfully yours,

G. E. F.

ANSWER:—You probably will have to submit to another operation. The diseased bone should be removed—it will continue to discharge as long as it remains there. Medicines will do no good.

---

YPSILANTI, Mich., April 26, 1869.

T. S. UP DE GRAFF, M. D.,

*Dear Sir:*—I am truly gratified in having the privilege of subscribing for your medley (the BISTOURY.) From its form, (pamphlet,) I hope it is to be a permanent publication. If it could get a general circulation, and be generally read, much good might result to the sick. Your exposure of quackery must have an influence upon the sickly fools who are their victims.

Inclosed please find the price of Vol. 5, which direct to J. W. B., M. D.

—Thanks, Doctor; we will endeavor to make it "red-hot" (as Brick Pomeroy says,) for all manner of swindlers.

---

ELMIRA, June 1, 1869.

DR. UP DE GRAFF:

In the country, a few days since, I was obliged to tell the husband of a poor consumptive that his wife must die very soon—might die any day. Said he: "I wrote to Dr. Leonidas Hamilton, of New York, a careful account of the case, and he said she could be cured, and he would send the medicine for fifteen dollars and a-*half*."

In Rochester, some years ago, a man was selling some form of supporter for twenty-seven dollars and a *fraction*. Are not these men Charlatans? The unlooked for ear-mark of fraud is in the *odd* sum, or *fractional* part of a dollar, which makes the exorbitant charge *seem* to be based upon some accurate estimate of cost. P. H. H.

REPLY:—We see no difference in the proceedings of Dr. Hamilton and "Rev. Henry A. Wilson" or "Dr. John B. Ogden." The last two advertise to send formulæ for the cure of consumption and spermatorrhœa, free of charge, and finally manage to secure from five to fifteen dollars from their victims for worthless medicines(?) Should any one but Dr. Hamilton do a deed of that sort, he would, at once, be denounced a quack.

---

THE VEHICLE.—A poor woman, whose husband kept his bed with a lingering illness, went to a neigboring physician, who kindly gave her a prescription, and directed her to the chemist to get it made up. When finished, the man stupidly wrote on it, "to be taken in a vehicle." Now, "vehicle" was a word so far beyond the good woman's capacity, she thought no one but the clergyman of the parish learned enough to explain its meaning. Not showing him the label, she merely asked him, "what was a vehicle?" He replied a phæton, a curricle, a landau, a whiskey, or a wheelbarrow. The last of these terms exactly suited her comprehension, and she returned home vastly pleased, where she actually made her husband rise, come down stairs, get into a wheelbarrow, take his physic, and go to bed again.—*Harper's Bazar.*

